# Optimizing HIV testing services in sub‐Saharan Africa: cost and performance of verification testing with HIV self‐tests and tests for triage

**DOI:** 10.1002/jia2.25237

**Published:** 2019-03-25

**Authors:** Jeffrey W Eaton, Fern Terris‐Prestholt, Valentina Cambiano, Anita Sands, Rachel C Baggaley, Karin Hatzold, Elizabeth L Corbett, Thoko Kalua, Andreas Jahn, Cheryl C Johnson

**Affiliations:** ^1^ Department of Infectious Disease Epidemiology Imperial College London London United Kingdom; ^2^ Department of Global Health and Development London School of Hygiene and Tropical Medicine London United Kingdom; ^3^ Institute for Global Health University College London London United Kingdom; ^4^ Essential Medicines and Health Products Department World Health Organization Geneva Switzerland; ^5^ Global HIV and Hepatitis Department World Health Organization Geneva Switzerland; ^6^ Population Services International Johannesburg South Africa; ^7^ Clinical Research Department London School of Hygiene and Tropical Medicine London United Kingdom; ^8^ Malawi Liverpool Wellcome Trust Clinical Research Programme Blantyre Malawi; ^9^ Department of HIV/AIDS Ministry of Health Lilongwe Malawi; ^10^ International Training and Education Center for Health (I‐TECH) Lilongwe Malawi

**Keywords:** HIV, HIV testing, HIV self‐testing, Retesting, ART initiation, Quality

## Abstract

**Introduction:**

Strategies employing a single rapid diagnostic test (RDT) such as HIV self‐testing (HIVST) or “test for triage” (T4T) are proposed to increase HIV testing programme impact. Current guidelines recommend serial testing with two or three RDTs for HIV diagnosis, followed by retesting with the same algorithm to verify HIV‐positive status before anti‐retroviral therapy (ART) initiation. We investigated whether clients presenting to HIV testing services (HTS) following a single reactive RDT must undergo the diagnostic algorithm twice to diagnose and verify HIV‐positive status, or whether a diagnosis with the setting‐specific algorithm is adequate for ART initiation.

**Methods:**

We calculated (1) expected number of false‐positive (FP) misclassifications per 10,000 HIV negative persons tested, (2) positive predictive value (PPV) of the overall HIV testing strategy compared to the WHO recommended PPV ≥99%, and (3) expected cost per FP misclassified person identified by additional verification testing in a typical low‐/middle‐income setting, compared to the expected lifetime ART cost of $3000. Scenarios considered were as follows: 10% prevalence using two serial RDTs for diagnosis, 1% prevalence using three serial RDTs, and calibration using programmatic data from Malawi in 2017 where the proportion of people testing HIV positive in facilities was 4%.

**Results:**

In the 10% HIV prevalence setting with a triage test, the expected number of FP misclassifications was 0.86 per 10,000 tested without verification testing and the PPV was 99.9%. In the 1% prevalence setting, expected FP misclassifications were 0.19 with 99.8% PPV, and in the Malawi 2017 calibrated setting the expected misclassifications were 0.08 with 99.98% PPV. The cost per FP identified by verification testing was $5879, $3770, and $24,259 respectively. Results were sensitive to assumptions about accuracy of self‐reported reactive results and whether reactive triage test results influenced biased interpretation of subsequent RDT results by the HTS provider.

**Conclusions:**

Diagnosis with the full algorithm following presentation with a reactive triage test is expected to achieve PPV above the 99% threshold. Continuing verification testing prior to ART initiation remains recommended, but HIV testing strategies involving HIVST and T4T may provide opportunities to maintain quality while increasing efficiency as part of broader restructuring of HIV testing service delivery.

## 1 Introduction

Substantial scale‐up of HIV testing services (HTS) has contributed to tremendous progress towards global targets to diagnose 90% of people with HIV by 2020. In 2017, PEPFAR alone conducted more than 85 million HIV tests 1. Despite this scale‐up, an estimated 25% of people with HIV remain unaware of their status 2.

Striving for these ambitious targets for HIV diagnosis, while also seeking increases in the efficiency and effectiveness of services, has stimulated innovative approaches to providing HTS. Recent forecasts suggest the HIV response is not on track to achieve the 90‐90‐90 testing and treatment targets unless significant investments are made 3, and there are increases in effectiveness and efficiency of services. The expanded volume of HIV testing and depletion of undiagnosed persons has increased the marginal testing cost per HIV‐positive person identified. Static donor investment has also added pressures to implement more “cost‐effective” testing approaches. As a result, many countries are looking for innovative ways to continue to scale‐up HIV testing, while maximizing effectiveness and efficiency and maintaining quality.

**Figure 1:**
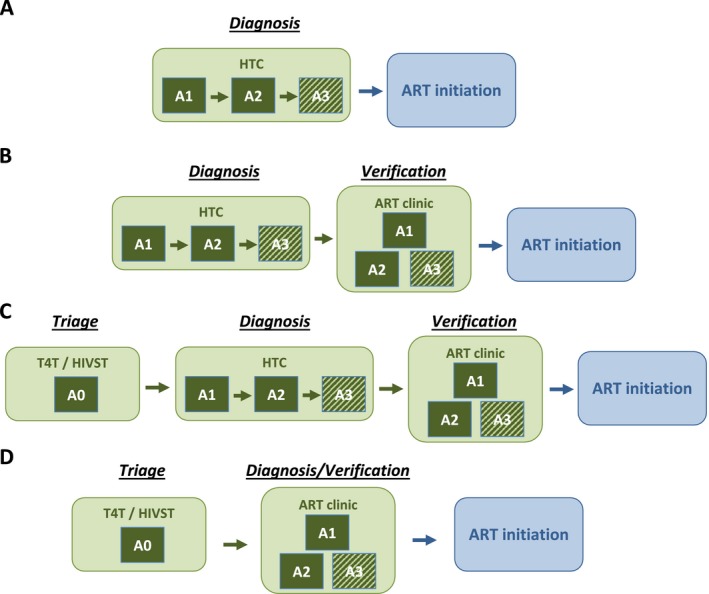
Simplified flow diagram for alternative HIV testing and diagnosis strategies prior to ART initiation (**A** and **B**) represent current “status quo” HIV testing strategies without and with verification testing prior to ART initiation, respectively. (**C** and **D**) illustrate potential testing strategies for clients presenting to HIV testing services (HTS) following a single reactive RDT through HIV self‐testing or test for triage modalities. In (**C** and **D**), the “A0” assay represents a single RDT, either HIVST or T4T, applied before referral to HIV testing services for testing and diagnosis with the full diagnostic algorithm. Assays “A1,” “A2,” and “A3” represent HIV antibody rapid diagnostic test (RDT) conducted in serial comprising a testing algorithm in a setting using a 2‐test strategy or 3‐test strategy. The “A3” assay is shaded to indicate that this assay is only applied in a setting using a 3‐test strategy for HIV diagnosis (recommended for prevalence <5%). HIV diagnosis is established only if all two/three serial RDTs are reactive. Discordant results (A1 reactive/A2 non‐reactive) should be re‐tested using the same two assays; if they remain discrepant, the result is reported as HIV‐inconclusive and the client is retested in 14 days. Full details of the flow and reporting of results in the case of discrepant results are described in 4. In our simplified simulation, it is assumed that discrepant results will be adjudicated correctly upon retesting, and thus FP misclassification only occurs if the results of all two or three assays are misclassified as reactive in serial. ART, anti‐retroviral therapy; HIVST, HIV self‐testing; HTC, HIV testing and counselling; RDT, rapid diagnostic test.

To establish a diagnosis of HIV infection, WHO Guidelines recommend using multiple independent serological assays (rapid diagnostic tests (RDTs) and enzyme immunoassays) 4. Each assay must demonstrate at least 99% sensitivity and 98% specificity. In settings where the prevalence among HTS clients is above 5%, guidelines recommend reactive results from two consecutive assays conducted serially to establish HIV infection, and three consecutive assays in settings with HIV prevalence below 5% (Figure 1A), ensuring a positive predictive value (PPV) of above 99% in all settings 4. If the results of the assays are discrepant, both assays are repeated. In the high prevalence setting (≥5%), if still discrepant, a third assay is applied. If the third assay is non‐reactive, the result is reported as HIV negative, while if reactive the result is reported as inconclusive to be retested in 14 days. In the low prevalence setting, all three assays must be reactive to establish HIV infection; if the first two are reactive and the third non‐reactive, the result is reported as inconclusive for re‐testing in 14 days.

Recent reports have described suboptimal quality of HIV testing and cases of HIV misdiagnosis 5, highlighting the importance of ensuring reliable and accurate HIV testing, alongside scale‐up. A recent systematic review identified the main reason for false‐positive (FP) HIV diagnosis was the use of incorrect or suboptimal testing strategies and algorithms in facilities 6. To mitigate misclassification of HIV status (often due to human error), WHO recommends re‐testing with the full diagnostic algorithm by an independent provider to “verify” HIV‐positive status immediately prior to anti‐retroviral therapy (ART) initiation (Figure 1B) 4. Recent analyses estimated that additional verification testing prior to ART initiation is highly cost‐saving 6, 7, in addition to being good public‐health practice, but many countries are yet to implement this approach 8.

HIV self‐testing (HIVST) and “test for triage” (T4T) are two testing modalities that both involve provision of a single HIV RDT, referred to as an “A0” (assay 0) test, either by oneself (HIVST) or a lay‐provider in a community‐based setting (T4T). Clients with reactive A0 RDT results are linked to the health system for testing with the full national testing algorithm to confirm HIV‐positive status. Such strategies offer opportunities to reach those with a single RDT who may not otherwise test and then promote to further testing and treatment linkage for those with a reactive test result 4, 9. HIVST has been highlighted as an effective way to increase uptake and frequency of testing in high risk populations 10. These approaches offer the opportunity to improve quality and efficiency in the health system, including fast‐tracking those with reactive results to care and those who are negative to prevention 4, 9. In the light of evidence suggesting suboptimal specificity of HTS 6 and recent evidence that HIVST is highly specific 11, many countries rolling out HIVST are considering whether additional verification testing is still required before ART initiation for people presenting to care following a reactive HIVST result.

This analysis considers whether patients with a single reactive A0 RDT, from either HIVST or T4T, must undergo the full testing strategy twice (1) for diagnosis and (2) for verification testing prior to ART initiation, or if is it adequate to initiate ART following an initial reactive A0 result followed by a diagnosis with the full testing strategy alone (i.e. without additional verification of HIV status at ART initiation, hereafter verification testing).

## 2 Methods

We used a simple probability model to calculate the expected levels of FP misclassification arising from HIV testing strategies that included an A0 RDT prior to presenting for HTS compared to current HIV testing strategies that do not include an A0 test. The model extends a previously developed model of WHO recommended HIV testing strategies, including verification testing, to incorporate a single RDT as an A0 T4T 6. All analyses were conducted in R version 3.5.0. An R script reproducing all analyses is provided as Data S1.

### 2.1 Testing strategies considered

Figure 1 presents the four testing strategies considered. The first two (A and B) are “status quo” HIV testing strategies without or with verification testing before ART initiation (Figures 1A and 1B respectively); WHO HTS Guidelines recommend testing including verification (Figure 1B). The HTS client is considered “diagnosed” following reactive results on two independent RDTs (A1 + A2) in a setting with prevalence above 5% or three independent RDTs (A1 + A2 + A3) in a setting with prevalence below 5%. Following diagnosis, the client is referred for HIV care and treatment at which point the full two‐test or three‐test HIV testing strategy is repeated to verify the HIV status (Figure 1B), followed by ART initiation if the HIV diagnosis is confirmed.

In the third strategy (Figure 1C), we considered that clients underwent an A0 test with a reactive result prior to presentation at HTS. Following this they proceed through the full diagnosis and verification before initiating ART. In the fourth strategy, we considered combining the “diagnosis” and “verification” stages for clients presenting for HTS following a reactive A0 test (Figure 1D). That is, they are initiated to ART following a single sequence of two or three reactive RDTs per the validated national testing algorithm.

### 2.2 Modelled scenarios and assumptions about RDT performance in diagnostic settings

As base scenarios, we considered a “high‐prevalence” setting using a two‐test strategy with 10% HIV prevalence among testing clients and a “low‐prevalence” setting employing a three‐test strategy with 1% HIV prevalence. Consistent with previous application of our model 6, we assumed 98% specificity of each RDT in the algorithm, which is the minimum specificity required for WHO prequalification 12. We further assumed a 20% probability that a FP misclassification on one RDT would also be misclassified on the subsequent independent RDT 6. This is due to potential correlated exogenous factors that might influence correlated FP classification errors, such as environmental conditions or user errors affecting the outcome of both tests. The specificity for the overall testing algorithm (A1 + A2 or A1 + A2 + A3) is calculated as one minus the probability that both or all three assays are reactive given the true status is HIV negative:

$$\mathrm{spe}c_{2-\mathrm{test}}=1-\left(1-\mathrm{spe}c_{A1}\right)\cdot \left(c+\left(1-c\right)*\left(1-\mathrm{spe}c_{A2}\right)\right)$$

$$\begin{array}{cc} & {\text{spec}}_{3-\mathrm{test}}=1-\left(1-{\text{spec}}_{A1}\right)\cdot \left(c+\left(1-c\right)\cdot \left(1-{\mathrm{spec}}_{A1}\right)\right.\\ & \left.\;\;\cdot \left(c+\left(1-c\right)\cdot \left(1-{\mathrm{spec}}_{A3}\right)\right)\right) \end{array}$$

where spec_A*x*_ = 0.98 is the specificity for each individual assay and c = 0.2 is the additional probability that a FP misclassification one on RDT results in a misclassification on the next RDT in the algorithm. These assumptions imply overall testing algorithm specificity of spec_2‐test_ = 99.57% for the two‐test strategy and spec_3‐test_ = 99.91% for the three‐test strategy.

We considered a third scenario indicative of the performance of the national HIV testing programme of Malawi in 2017. Malawi currently uses a two‐test strategy and has conducted verification testing prior to ART initiation since 2011. The national HIV prevalence among adults in 2017 was 10% 13, the positivity among HTS clients was 4% across all testing modalities including health facilities, non‐health facility venues, mobile testing, and community‐based testing 14. According to 2017 verification testing records, of the 174,078 clients testing positive and undergoing verification, 1481 (1%) were subsequently found to be HIV negative of the 174,078 testing HIV positive and undergoing verification 14. The prevalence among testers of 4% and PPV of 99% imply that the specificity for the two‐test algorithm is 99.96%.

### 2.3 Assumptions about performance of A0 tests

We assumed a specificity of 98% for A0 tests conducted via HIVST or T4T modalities as a base assumption and varied specificity from 90% to 100% in sensitivity analyses 11. In our base analysis, we assumed that the outcome of the A0 test does not affect accuracy of subsequent diagnostic testing conducted by an HTS provider. However, in sensitivity analysis we considered the potential effect of knowledge of the A0 test result influencing reader error resulting in FP misclassification by the HTS provider. In sensitivity analysis, we modelled a probability ranging from 0% to 20% that the A1 test would be misclassified for an HIV‐negative individual presenting for HTS following a FP A0 triage test result. We report results focused on 0% and 5% probability of reader error.

### 2.4 Cost assumptions

We assumed a cost of $7 per client for verification retesting with the three‐test strategy and $5 per client for verification retesting with the two‐test strategy, informed by HIV testing cost data typical for sub‐Saharan Africa 15. We estimated the discounted lifetime ART cost of $3000. This was based on an annual cost of $150 per year in sub‐Saharan Africa including ARV commodities, diagnostics, and clinical monitoring, and service delivery for stable ART patients 16 over 30 years life expectancy discounted at 3% per annum 17 assuming no loss to follow‐up. All costs were considered in 2016 US dollars.

### 2.5 Analysis

For each testing strategy and scenario (“high prevalence”—10%, “low prevalence”—1%, “Malawi 2017”—4%), we calculated three outcomes of interest:

1. The expected number of FP misclassifications per 10,000 HIV‐negative persons tested.
2. The expected PPV for the overall testing strategy, that is, the probability that a person initiated on ART is truly HIV positive.
3. The expected cost per FP person identified through verification re‐testing compared to the expected lifetime cost of ART.

For the calculation of PPV, we conservatively assumed a sensitivity of 90% among HIV‐positive clients, such that:

$$\mathrm{PPV}=\frac{\mathrm{sens}\cdot \mathrm{prev}}{\mathrm{sens}\cdot \mathrm{prev}+\left(1-\mathrm{spe}c_{\mathrm{strgy}}\right)\cdot \left(1-\mathrm{prev}\right)}$$

where spec_strgy_ is the specificity of the overall testing strategy including any A0 test or verification testing. We considered testing strategies “acceptable” if the PPV for the overall testing strategy was above the 99% threshold defined by the WHO Guidelines 15.

We considered additional verification testing “cost‐efficient” if the cost per FP misclassification identified was less than the expected lifetime ART cost of $3000. The total cost of verification testing was the cost per client for verification testing ($5 or $7 depending on 2‐test or 3‐test algorithm) times the number of clients classified as HIV positive before verification testing:

$$\begin{array}{cc} \left[\mathrm{verification}\;\mathrm{cost}\right] & =\left[\mathrm{cost}\;\mathrm{per}\;\mathrm{verification}\;\mathrm{client}\right]\\ & \;\cdot \left(\mathrm{sens}\cdot \mathrm{prev}+\left(1-\mathrm{spe}c_{\mathrm{no}-\mathrm{verif}}\right)\cdot \left(1-\mathrm{prev}\right)\right) \end{array}$$

The expected number of false positive cases identified through verification testing was calculated as the number of negative clients testing times the specificity of the strategy with verification testing minus the specificity of the same strategy without verification testing:

$$\left[\text{FP identified}\right]=\left(1-\mathrm{prev}\right)\cdot \left({\text{spec}}_{w/\mathrm{verif}}-{\text{spec}}_{\mathrm{no}-\mathrm{erif}}\right)$$

The cost per FP identified was the ratio of total verification cost divided by the number of FP identified.

## 3 Results

### 3.1 Rates of false positive misclassification

#### 3.1.1 Scenario 1: High prevalence (10%)

In scenario 1, with 10% HIV prevalence and using the two‐test strategy, the “status quo” scenario of HIV diagnosis following reactive A1 and A2 RDTs without additional verification testing (Figure 1A) resulted in 43.2 FP misclassifications per 10,000 HIV‐negative persons tested (Table 1). Implementing verification testing by retesting using the full testing strategy (Figure 1B) reduced the number of misclassifications to 0.64. The PPV increased from 95.9% to 99.9%.

**Table 1:** Results for number of false positive misclassifications, PPV, and cost per FP identified for alternative scenarios and testing strategies

	10% prevalence			1% prevalence			Malawi 2017^a^		
	Status Quo	With A0	A0, 5% A1 error	Status Quo	With A0	A0, 5% A1 error	Status Quo	With A0	A0, 5% A1 error
Testing Strategy	Two‐test			Three‐test			Two‐test		
Algorithm specificity	99.57%			99.91%			99.96%		
Verification testing cost	$5			$7			$5		
False positive misclassifications per 10,000 HIV negative persons tested									
No verification^b^	43.2	0.86	2.98	9.3	0.19	0.64	4.18	0.08	2.10
With verification^c^	0.64	0.01	0.04	0.03	0.001	0.002	0.04	0.001	0.02
PPV									
No verification^b^	95.86%	99.91%	99.70%	90.69%	99.80%	99.30%	98.90%	99.98%	99.44%
With verification^c^	99.94%	>99.99%	>99.99%	99.97%	>99.99%	>99.99%	99.99%	>99.99%	99.99%
Cost per FP identified^d^	$123	$5,880	$1,708	$75	$3,428	$999	$460	$22,743	$909

FP, false‐positive; PPV, positive predictive value. ^a^Prevalence among HIV testing clients was 4% in Malawi in 2017. HIV prevalence among all adults was approximately 10%. ^b^“No verification” corresponds to strategy in Figure 1A under “status quo” scenario and Figure 1D for “with A0” scenarios. ^c^“With verification” corresponds to Figure 1B under “status quo” scenario’ and Figure 1C under “with A0” scenarios.^d^Cost per FP identified through verification testing compared to no verification testing. Cost is calculated as the expected number of verification tests conducted (sens × prev + (1 − spec) × (1 − prev)) times the cost per verification test divided by the number of FP cases identified through verification testing.

When HTS clients had a reactive A0 triage test prior to full diagnosis at HTS, the expected number of misclassifications was only 0.86 and the PPV was 99.9% without additional verification testing (Figure 1D). Additional verification testing (Figure 1C) reduced the number of misclassifications to 0.01. When we assumed that a false‐reactive A0 RDT may induce a 5% probability of reader error of the A1 RDT at HTS, the expected number of FP for the diagnosis without additional verification increased to 3.0, but the PPV was 99.7%, remaining well above the 99% target threshold.

#### 3.1.2 Scenario 2: Low prevalence (1%)

In scenario 2, with 1% HIV prevalence and using the a three‐test strategy, the expected number of FP misclassifications was lower for all strategies due to the inclusion of the third RDT, but the PPV was also slightly lower due to the lower prevalence (Table 1). The number of FP for HIV diagnosis following a single application of the three‐test algorithm was 9.3 and the PPV was 90.7%. For clients presenting with a reactive A0, the number of FP reduced to 0.2 and the PPV was 99.8%. Assuming a 5% error for the A1 test following a false‐reactive A0 changed the expected FP to 0.6 and the PPV to 99.3%, still above the 99% threshold.

#### 3.1.3 Scenario 3: Malawi 2017 (4% positivity; 99% PPV)

In the scenario based on programmatic data from Malawi in 2017, in which HIV positivity among HTS clients was 4% and the two‐test strategy performed with a 99% PPV, the expected number of FP misclassifications was 4.2 per 10,000 HIV‐negative persons tested. The number of FP reduced to 0.08 with a 99.98% PPV for clients presenting with a reactive A0, or 2.1 FP and a 99.5% PPV when we assumed a 5% reader error for the A1 RDT.

### 3.2 Cost per false positive identified

Without A0 triage testing before presentation for HTS (“status quo” scenario), additional verification testing was clearly cost‐efficient. The estimated cost per FP misclassification identified was $123, $75, and $460 for the “high prevalence,” “low prevalence” and “Malawi 2017” scenarios respectively (Table 1), which compared very favourably to the $3000 expected lifetime cost had the misclassified client been initiated on ART. When clients presented for HTS following a reactive A0 RDT, the cost per FP identified was $5880 in the base case 10% prevalence setting, $3428 in the 1% prevalence setting, and $22,743 for the Malawi 2017 HTS assumptions. The cost per FP identified was lower when assuming 5% reader error in the A1 RDT at $1708, $999, and $909 respectively, but still markedly higher than the cost per FP identified associated with verification testing in the absence of the A0 triage test.

### 3.3 Sensitivity analysis of A0 specificity

**Figure 2:**
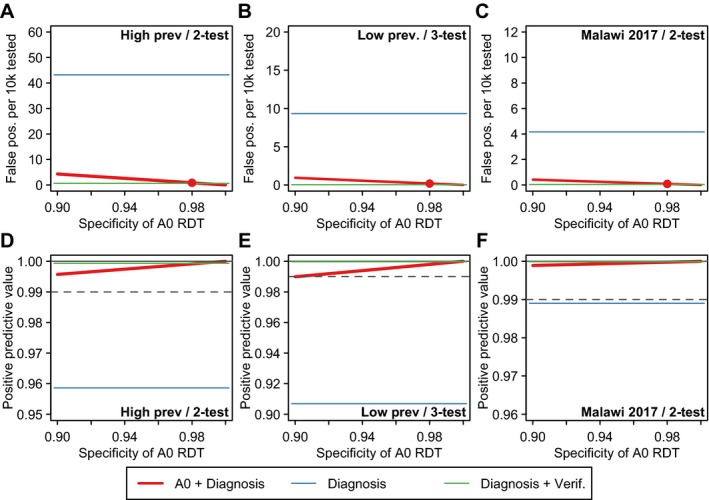
Sensitivity analysis of assumptions the specificity of the A0 RDT on the expected number of false positive misclassifications per 10,000 HIV negative persons tested (**A** to **C**) and the positive predictive value (PPV) of the overall testing strategy, conservatively assuming 90% sensitivity (**D** to **F**) Red line illustrates scenario in which clients present to HTS following a reactive test and undergo the national HIV testing algorithm once (Figure 1D). Red points mark the assumed 98% specificity assumed in the base‐case analysis. For benchmarking, the blue horizontal line indicates the results for status quo HIV testing without verification testing (Figure 1A) and the green line indicates status quo testing with verification testing (Figure 1B). For PPV results (**D** to **F**), the grey dashed line indicates the 99% PPV threshold recommended by WHO Consolidated HIV Testing Guidelines. RDT, rapid diagnostic test.

**Figure 3:**
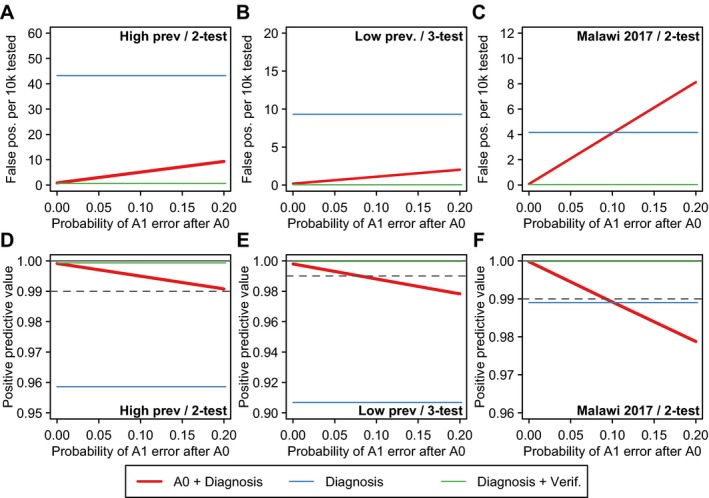
Sensitivity analysis about the probability of excess reader error for A1 test if presenting following a false‐reactive result of the A0 RDT. (**A** to **C**) illustrate the effect on the expected number of false positive misclassifications per 10,000 HIV negative persons tested and (**D** to **F**) illustrate the positive predictive value (PPV) of the overall testing strategy, conservatively assuming 90% sensitivity Red line illustrates scenario in which clients present to HTS following a reactive A1 test and undergo the national HIV testing algorithm once (Figure 1D). For benchmarking, the blue horizontal line indicates the results for status quo HIV testing without verification testing (Figure 1A) and the green line indicates status quo testing with verification testing (Figure 1B). For PPV results (**D** to **F**), the grey dashed line indicates the 99% PPV threshold recommended by WHO Consolidated HIV Testing Guidelines. HTS, HIV testing services; RDT, rapid diagnostic test.

Figure 2 considers the sensitivity of these conclusions to assumptions about the specificity of the A0 triage test, assumed to be 98% in the base‐case analysis in Table 1. For specificity values ranging between 90% and 100%, the expected number of FP cases is higher and PPV lower for lower A0 specificity, but in all cases the PPV is well above the 99% PPV target threshold. Figure 3 considers the sensitivity to the assumed probability of reader error misclassification of the A1 test following attendance with a reactive A0 triage test, which was assumed to be 0% or 5% in Table 1. Higher probabilities of reader error associated with a FP A0 result reduced the performance of the overall testing strategy, and at high levels of error additional verification testing may be needed to meet the 99% PPV target threshold. In the Malawi 2017 scenario, in which the specificity of the A1 test was higher than the 98% assumed in the baseline scenario, HTS performance could be worse than the status quo without verification testing if a FP A0 test resulted in greater than 10% reader error in A1 test results, and this threshold would vary depending on the attained specificity of the A0 test.

## 4 Discussion

The recommendation for verification of HIV status before ART initiation has been increasingly adopted to ensure the fidelity of the HIV testing and ART programmes and avoid future costs and ramifications associated with inadvertently initiating HIV‐negative persons on lifelong ART. Previous studies have highlighted that retesting may be particularly important considering reports of poor quality testing and low uptake of WHO‐recommended HIV testing strategies and algorithms 5, 18, 19, 20, 21. Policy analysis from 2015 suggested fewer than 20% of reporting countries had a national testing strategy and algorithm that was in full alignment with WHO guidelines 8.

In this analysis, we considered whether such additional verification testing is required and cost‐efficient for clients who already underwent diagnostic testing to confirm their HIV status in HTS following a reactive triage test. Taken together, our analysis suggests triage test approaches, including HIVST, can potentially result in lower FP HIV misclassification in settings not implementing verification testing prior to ART. We find that the rate of FP misclassification is expected to be very low for persons presenting with a reactive HIVST confirmed by diagnostic testing and the PPV is expected to be well above 99% without additional verification testing. This is the case even with base assumptions about the accuracy of HTS that appear conservative compared to programmatic data about the current performance of HIV testing in programmatic settings. Given the very small number of clients expected to be identified as FP, verification testing may not be cost‐efficient relative to the lifetime costs of ART for FP persons.

Even with imperfect specificity of HIVST, the expected number of FP among persons presenting for HTS following a reactive HIVST is far lower than would be expected amongst a population of HTS clients who had not undergone triage testing. Most basically, this conclusion reflects the difference in the prior probability that a client is truly HIV positive before presenting to HTS. In the absence of triage testing, with a true HIV positivity of 5% among those testing, 95% of clients will be truly HIV negative, leaving a large pool among which a FP misclassification could occur. Among 100 HTS clients presenting following a reactive HIVST with 98% specificity, only 2% would be truly HIV negative, a much smaller group among whom a FP misclassification could occur.

Our analysis has several limitations and the findings should be considered in the light of important uncertainties about key assumptions. First, findings were sensitive to whether we assumed that presenting with a reactive A0 test might bias the provider in interpreting results of subsequent A1 RDT results. Currently, even in relatively high HIV prevalence settings, the large majority (>95%) of HTS clients are classified as HIV negative following a single RDT. This would change for a provider seeing a large number of clients referred to care following a reactive HIVST or T4T. This could change the prior expectations of the provider about the likely outcome of the test and subtly bias the interpretation of inconclusive test results. However, to our knowledge, evidence is not yet available to evaluate whether this occurs. We consider this a high priority evidence gap for further research as HIVST scales up.

Second, our analysis takes a narrow perspective on the potential costs and consequences of FP misclassification by considering only the costs to the health system associated with lifetime ART for a misdiagnosed client. Costs and adverse consequences born by clients may be substantially greater, including unnecessary care and treatment, consequences for family, marriages, and relationships, potential adverse effects of ART. Without capturing the full health and quality of life consequences of HIV misclassification, we are not able to undertake cost‐effectiveness analysis to benchmark investments in verification testing against other potential allocation of health resources. More broadly, cases of FP misclassification may serve to undermine confidence and engagement in the health system outstripping the economic costs of unnecessary treatment.

Third, we considered only the risk of false positive diagnosis amongst HIV‐negative clients. Ensuring highly accurate HIV diagnosis is paramount for HIV testing services. Evidence suggests that rates of false negative misclassification in both traditional HTS and HIVST are also higher than would be expected given 99% sensitivity required for WHO prequalification 11, 22. Quantifying the rates, reasons, and consequences of false negative diagnosis is an important area for further implementation research and modelling.

These modelling results need to be considered in the light of practical implementation issues. Although the expected number of FP misclassifications identified through verification testing was low for persons presenting following HIVST, it is not recommended to discontinue verification testing for these clients in settings where verification testing is already in place, working well, and achieving results. It will be important to review data from settings where this new testing strategy is used before suggesting changing current recommended practice. For example, in 2015, Malawi was one of few countries implementing WHO recommended testing strategies and verification testing among people with HIV prior to starting ART 4, 8. These efforts combined with updated guidelines and re‐training of testers, decreased HIV‐negative test results following an initial HIV‐positive diagnosis from 7% to 1% between 2014 and 2016 14. Additional studies have highlighted the role of retesting, alongside validation of national algorithms, to ensure quality 18, 20.

The HIV testing resources required for verification testing is a small proportion of the overall HIV testing resources, considering that the very large majority of HTS clients will be classified as HIV negative from the first assay in the HIV testing algorithm 6. This will especially be the case as positivity and number of new diagnoses decline as a share of all those tested. Currently HIVST is not available at national scale in most settings, and proposing different models for diagnosis, verification, and treatment initiation for these few HIVST clients may potentially increase fragmentation of HTS, which could increase opportunities for errors. Broader changes in future HTS delivery may reconsider the role of verification *vis‐à‐vis* the “test‐for‐triage” model in which a single RDT is applied at the first engagement with HIV testing services, following which clients are referred to HIV care and treatment facilities for full diagnostic testing with the full national HIV testing algorithm. For example, embracing the “test‐for‐triage” model across all HIV testing modalities, whether facility‐based or community‐based may simplify and streamline the provision of HTS, and harmonize client flow for HIVST clients with those engaging through other modalities. Such approaches should be considered and evaluated in programmatic settings.

Programmatically, it may be most advantageous to promote T4T and HIVST as simplified initial screening tests on a large scale and deliver quality‐assured verification testing directly before ART initiation at health facilities, cutting out the need for parallel “intermediate” confirmation testing at peripheral testing sites.

## 5 Conclusions

Following WHO testing strategies with verification testing prior to ART initiation is recommended and should be continued. T4T and HIVST approaches could potentially improve accuracy and quality of HTS in settings not implementing diagnostic testing followed by repeated verification testing prior to ART. T4T followed by diagnosis with full national testing algorithm is expected to deliver accurate results above WHO benchmarks for PPV of at least 99%, so long as the quality and specificity of HTS remains similar to current programmatic performance. While HIVST scale‐up may render verification testing before ART less necessary in high quality programmes, selectively discontinuing full diagnosis with the national testing algorithm before verification testing for a subset of clients who present following reactive A0 test must be considered agains the risk risks of additional complexity and potential for increased user and provider error. T4T and HIVST may provide an opportunity to restructure HTS delivery and quality assurance systems which should be explored further and evaluated in programmes to guide future policy.

## Competing interests

We have no competing interests to declare.

## Authors’ contributions

JWE and CJ drafted initial manuscript with inputs from all authors. All authors contributed to the final manuscript.

## Supporting information


**Data S1.** R code for reproducing analyses.
