# Supplementary material for: Optimizing HIV testing services in sub‐Saharan Africa: cost and performance of verification testing with HIV self‐tests and tests for triage
Source: J Int AIDS Soc. 2019 Mar 25;22(Suppl Suppl 1):e25237. doi: 10.1002/jia2.25237 (PMC6545556; doi:10.1002/jia2.25237)
Supplement: Supplementary file 1 — Data S1. R code for reproducing analyses. [file JIA2-22-e25237-s001.docx]

#' Definitions:

#' - ass_spec: specificty for a single assay

#' - alg_spec: specificity for diagnosis algorithm (2 or 3 tests)

#' - sgy_spec: specificity for an overall tesitng strategy

#' - corr: correlation of assays in the same algorithm

#' - corr_alg: correlation of previous outcome and first test of new alg

#' - corr_a0: correlation of A0 and HTS testing

#' Solve for individual assay specificity from algorithm specificity

#' accounting for correlated error between A1 and A2 assay

solve_ass_spec2 <- function(alg_spec2, corr){

## solution to 1 - p * (c + (1-c) * p) = a

## where p = 1 - ass_spec; c = corr; a = alg_spec

c <- corr

a <- alg_spec2

p <- (- c + sqrt(c^2 - 4*(1-c)*(a-1))) / (2*(1-c))

return(1 - p)

}

## Calculate specificity for Malawi 2017 testing programme performance

ass_spec <- 0.98

corr <- 0.2

alg_spec2_mw <- 1 - (.04*.01) / (0.96)

ass_spec2_mw <- solve_ass_spec2(alg_spec2_mw, corr)

#' Calculate specificity of overall testing strategy (Figure 1 A-D)

#'

#' @param alg value 2 or 3 for 2-test or 3-test strategy

#' @param verif TRUE or FALSE indicating whether verification testing

#' @param a0 TRUE of FALSE indicating whether triage testing

#'

calc_sgy_spec <- function(alg = 2,

verif = FALSE,

a0 = FALSE,

ass_spec = 0.98,

a0_spec = 0.98,

corr = 0.2,

corr_alg = 0.05,

corr_a0 = 0.0){

## Probability of error on A1

p <- 1 - ass_spec

## Probability of error on second or third assay

p23 <- corr + (1-corr)*p

## Probability of error on first HTS

if(a0)

p1h <- corr_a0 + (1-corr_a0) * p

else

p1h <- p

## Probability of error on first verification test

p1v <- corr_alg + (1-corr_alg) * p

alg_p <- p1h * p23 ^ (alg - 1)

if(verif)

alg_p <- alg_p * p1v * p23 ^ (alg - 1)

if(a0)

alg_p <- (1 - a0_spec) * alg_p

1 - alg_p

}

## Prevalence for three settings considered

prev3 <- 0.01

prev2 <- 0.1

prev_mw <- 0.04

## Baseline assumptions: assay properties

spec <- 0.98

corr <- 0.2

corr_alg <- 0.05

#' Calculate PPV of testing strategy

calc_ppv <- function(prev, spec, sens=1.0){

sens * prev / (sens * prev + (1 - prev) * (1 - spec))

}

#' Calculate retesting costs

calc_verif_cost <- function(prev, cost, spec){

cost * prev * (1-spec)*(1-prev)

}

#' # Results

#'

#' Base case results

scen <- rbind(expand.grid(scen = "high",

prev = 0.1,

alg=2,

ass_spec = 0.98,

verif=c(FALSE, TRUE),

a0=c(FALSE, TRUE),

corr_a0 = c(0, 0.05)),

expand.grid(scen = "low",

prev = 0.01,

alg=3,

ass_spec = 0.98,

verif=c(FALSE, TRUE),

a0=c(FALSE, TRUE),

corr_a0 = c(0, 0.05)),

expand.grid(scen = "mw17",

prev = 0.04,

alg=2,

ass_spec = ass_spec2_mw,

verif=c(FALSE, TRUE),

a0=c(FALSE, TRUE),

corr_a0 = c(0, 0.05)))

scen$sgy_spec <- with(scen,

mapply(calc_sgy_spec,

alg = alg,

ass_spec = ass_spec,

verif = verif,

a0 = a0,

corr_a0 = corr_a0))

## Number of false positive per 10,000 HIV negative tested

scen$fp <- 1e4 * (1 - scen$sgy_spec)

## PPV for 90% sensitivity

scen$ppv <- calc_ppv(scen$prev, scen$sgy_spec, 0.9)

## Cost per false-positive identified

library(reshape2)

cost_per_fp <- dcast(scen, scen + prev + alg + ass_spec + a0 + corr_a0 ~ verif, value.var="sgy_spec")

cost_per_fp$testcost <- ifelse(cost_per_fp$alg == 2, 5, 7)

cost_per_fp$verifcost <- with(cost_per_fp, testcost * (0.9 * prev + (1 - prev) * (1 - `FALSE`)))

cost_per_fp$fp_identified <- with(cost_per_fp, (1 - prev)*(`TRUE` - `FALSE`))

cost_per_fp$cost_per_fp <- cost_per_fp$verifcost / cost_per_fp$fp_identified

## Lifetime ART cost

art_per_year <- 150

discount_rate <- 0.03

life_expectancy <- 30

lifetime_art_cost <- art_per_year * (1 - exp(-discount_rate * 30))/discount_rate

###################

#### Table 1 ####

###################

write.csv(dcast(melt(scen, id.vars=c("scen", "prev", "alg", "ass_spec", "verif", "t4t", "corr_t4t")),

variable + verif ~ scen + t4t + corr_t4t, value.var="value"),

"table1.csv")

dput(subset(rdhs::dhs_data(indicatorId = "HA_HIVP_W_HIV", countryId = "MW", surveyYear = "2015", breakdown = "all"), CharacteristicCategory == "Age (5-year groups)")$Value/100)

################################

#### Sensitivity analysis ####

################################

library(RColorBrewer)

#### Sensitivity to specificity of A1

quartz(h=4.2, w=6, pointsize=8)

par(oma=c(0, 0.5, 0, 0))

layout(rbind(1:3, 4:6, 7), h=c(5, 5, 1))

par(las=1, mar=c(2.5, 3, 1.5, 0.5), tcl=-0.25, mgp=c(2,0.5,0), cex=1)

##

cols <- brewer.pal(4, "Set1")

##

## Panel A

##

xx <- seq(0.9, 1.0, 0.005)

yy <- 1e4 * (1 - calc_sgy_spec(alg=2, FALSE, a0=TRUE, a0_spec=xx))

y1 <- 1e4 * (1 - calc_sgy_spec(alg=2, FALSE, FALSE))

y2 <- 1e4 * (1 - calc_sgy_spec(alg=2, TRUE, FALSE))

plot(xx, yy, type="l", ylim=c(0, 60), col=cols[1], lwd=2.5,

xlab="", ylab="False pos. per 10k tested", main="")

mtext("High prev / 2-test", 3, -1.05, adj=0.95, font=2)

title(xlab="Specificity of A0 RDT", line=1.5)

segments(0.898, y1, 1.002, col=cols[2], lwd=1)

segments(0.898, y2, 1.002, col=cols[3], lwd=1)

points(0.98, yy[which(xx==0.98)], col=cols[1], pch=19, cex=1.2)

mtext("A", 3, 0.3, at=0.87, font=2, cex=1.5)

##

xx <- seq(0.9, 1.0, 0.005)

yy <- 1e4 * (1 - calc_sgy_spec(alg=3, FALSE, a0=TRUE, a0_spec=xx))

y1 <- 1e4 * (1 - calc_sgy_spec(alg=3, FALSE, FALSE))

y2 <- 1e4 * (1 - calc_sgy_spec(alg=3, TRUE, FALSE))

plot(xx, yy, type="l", ylim=c(0, 20), col=cols[1], lwd=2,

xlab="", ylab="False pos. per 10k tested", main="")

mtext("Low prev. / 3-test", 3, -1.05, adj=0.95, font=2)

title(xlab="Specificity of A0 RDT", line=1.5)

segments(0.898, y1, 1.002, col=cols[2], lwd=1)

segments(0.898, y2, 1.002, col=cols[3], lwd=1)

points(0.98, yy[which(xx==0.98)], col=cols[1], pch=19, cex=1.2)

mtext("B", 3, 0.3, at=0.87, font=2, cex=1.5)

##

xx <- seq(0.9, 1.0, 0.005)

yy <- 1e4 * (1 - calc_sgy_spec(alg=2, FALSE, a0=TRUE,

ass_spec = ass_spec2_mw, a0_spec=xx))

y1 <- 1e4 * (1 - calc_sgy_spec(alg=2, FALSE, FALSE, ass_spec = ass_spec2_mw))

y2 <- 1e4 * (1 - calc_sgy_spec(alg=2, TRUE, FALSE, ass_spec = ass_spec2_mw))

plot(xx, yy, type="l", ylim=c(0, 12), col=cols[1], lwd=2,

xlab="", ylab="False pos. per 10k tested", main="")

mtext("Malawi 2017 / 2-test", 3, -1.05, adj=0.95, font=2)

title(xlab="Specificity of A0 RDT", line=1.5)

segments(0.898, y1, 1.002, col=cols[2], lwd=1)

segments(0.898, y2, 1.002, col=cols[3], lwd=1)

points(0.98, yy[which(xx==0.98)], col=cols[1], pch=19, cex=1.2)

mtext("C", 3, 0.3, at=0.87, font=2, cex=1.5)

##

## Panel B

##

par(mgp=c(2.2,0.5,0))

##

xx <- seq(0.9, 1.0, 0.005)

yy <- calc_ppv(0.1, calc_sgy_spec(alg=2, FALSE, a0=TRUE, a0_spec=xx), sens = 0.9)

y1 <- calc_ppv(0.1, calc_sgy_spec(alg=2, FALSE, FALSE), sens = 0.9)

y2 <- calc_ppv(0.1, calc_sgy_spec(alg=2, TRUE, FALSE), sens = 0.9)

plot(xx, yy, type="l", ylim=c(0.95, 1), col=cols[1], lwd=2.5,

xlab="", ylab="Positive predictive value", main="")

abline(h=c(0.99, 1.0), col="grey30", lty=c(2, 1))

mtext("High prev / 2-test", 1, -1.05, adj=0.95, font=2)

title(xlab="Specificity of A0 RDT", line=1.5)

segments(0.898, y1, 1.002, col=cols[2], lwd=1)

segments(0.898, y2, 1.002, col=cols[3], lwd=1)

mtext("D", 3, 0.3, at=0.87, font=2, cex=1.5)

##

xx <- seq(0.9, 1.0, 0.005)

yy <- calc_ppv(0.01, calc_sgy_spec(alg=3, FALSE, a0=TRUE, a0_spec=xx), sens = 0.9)

y1 <- calc_ppv(0.01, calc_sgy_spec(alg=3, FALSE, FALSE), sens = 0.9)

y2 <- calc_ppv(0.01, calc_sgy_spec(alg=3, TRUE, FALSE), sens = 0.9)

plot(xx, yy, type="l", ylim=c(0.9, 1), col=cols[1], lwd=2.5,

xlab="", ylab="Positive predictive value", main="")

abline(h=c(0.99, 1.0), col="grey30", lty=c(2, 1))

mtext("Low prev / 3-test", 1, -1.05, adj=0.95, font=2)

title(xlab="Specificity of A0 RDT", line=1.5)

segments(0.898, y1, 1.002, col=cols[2], lwd=1)

segments(0.898, y2, 1.002, col=cols[3], lwd=1)

mtext("E", 3, 0.3, at=0.87, font=2, cex=1.5)

##

xx <- seq(0.9, 1.0, 0.005)

yy <- calc_ppv(0.04, calc_sgy_spec(alg=2, FALSE, a0=TRUE,

ass_spec = ass_spec2_mw, a0_spec=xx), sens = 0.9)

y1 <- calc_ppv(0.04, calc_sgy_spec(alg=2, FALSE, FALSE, ass_spec = ass_spec2_mw), sens = 0.9)

y2 <- calc_ppv(0.04, calc_sgy_spec(alg=2, TRUE, FALSE, ass_spec = ass_spec2_mw), sens = 0.9)

plot(xx, yy, type="l", ylim=c(0.96, 1), col=cols[1], lwd=2.5,

xlab="", ylab="Positive predictive value", main="")

abline(h=c(0.99, 1.0), col="grey30", lty=c(2, 1))

mtext("Malawi 2017 / 2-test", 1, -1.05, adj=0.95, font=2)

title(xlab="Specificity of A0 RDT", line=1.5)

segments(0.898, y1, 1.002, col=cols[2], lwd=1)

segments(0.898, y2, 1.002, col=cols[3], lwd=1)

mtext("F", 3, 0.3, at=0.87, font=2, cex=1.5)

###

###

par(mar=c(0, 0, 0, 0))

plot(0, 0, type="n", bty="n", axes=FALSE)

legend("center", c("A0 + Diagnosis", "Diagnosis", "Diagnosis + Verif."), col=c(cols[1:3]), lty=c(1,1,1), lwd=c(4,2,2)/2, cex=1.0, inset=0.01, horiz=TRUE)

#### Sensitivity analysis: correlated error with A0 and A1

quartz(h=4.2, w=6, pointsize=8)

par(oma=c(0, 0.5, 0, 0))

layout(rbind(1:3, 4:6, 7), h=c(5, 5, 1))

par(las=1, mar=c(2.5, 3, 1.5, 0.5), tcl=-0.25, mgp=c(2,0.5,0), cex=1)

##

cols <- brewer.pal(4, "Set1")

xx <- seq(0, 0.2, 0.005)

##

## Panel A

##

yy <- 1e4 * (1 - calc_sgy_spec(alg=2, FALSE, a0=TRUE, corr_a0=xx))

y1 <- 1e4 * (1 - calc_sgy_spec(alg=2, FALSE, FALSE))

y2 <- 1e4 * (1 - calc_sgy_spec(alg=2, TRUE, FALSE))

plot(xx, yy, type="l", ylim=c(0, 60), col=cols[1], lwd=2.5,

xlab="", ylab="False pos. per 10k tested", main="")

mtext("High prev / 2-test", 3, -1.05, adj=0.95, font=2)

title(xlab="Probability of A1 error after A0", line=1.5)

segments(0.0, y1, 0.202, col=cols[2], lwd=1)

segments(0.0, y2, 0.202, col=cols[3], lwd=1)

mtext("A", 3, 0.3, at=-0.06, font=2, cex=1.5)

##

yy <- 1e4 * (1 - calc_sgy_spec(alg=3, FALSE, a0=TRUE, corr_a0=xx))

y1 <- 1e4 * (1 - calc_sgy_spec(alg=3, FALSE, FALSE))

y2 <- 1e4 * (1 - calc_sgy_spec(alg=3, TRUE, FALSE))

plot(xx, yy, type="l", ylim=c(0, 20), col=cols[1], lwd=2,

xlab="", ylab="False pos. per 10k tested", main="")

mtext("Low prev. / 3-test", 3, -1.05, adj=0.95, font=2)

title(xlab="Probability of A1 error after A0", line=1.5)

segments(0.0, y1, 0.202, col=cols[2], lwd=1)

segments(0.0, y2, 0.202, col=cols[3], lwd=1)

mtext("B", 3, 0.3, at=-0.06, font=2, cex=1.5)

##

yy <- 1e4 * (1 - calc_sgy_spec(alg=2, FALSE, a0=TRUE,

ass_spec = ass_spec2_mw, corr_a0=xx))

y1 <- 1e4 * (1 - calc_sgy_spec(alg=2, FALSE, FALSE, ass_spec = ass_spec2_mw))

y2 <- 1e4 * (1 - calc_sgy_spec(alg=2, TRUE, FALSE, ass_spec = ass_spec2_mw))

plot(xx, yy, type="l", ylim=c(0, 12), col=cols[1], lwd=2,

xlab="", ylab="False pos. per 10k tested", main="")

mtext("Malawi 2017 / 2-test", 3, -1.05, adj=0.95, font=2)

title(xlab="Probability of A1 error after A0", line=1.5)

segments(0.0, y1, 0.202, col=cols[2], lwd=1)

segments(0.0, y2, 0.202, col=cols[3], lwd=1)

mtext("C", 3, 0.3, at=-0.06, font=2, cex=1.5)

##

## Panel B

##

par(mgp=c(2.2,0.5,0))

##

yy <- calc_ppv(0.1, calc_sgy_spec(alg=2, FALSE, a0=TRUE, corr_a0=xx), sens = 0.9)

y1 <- calc_ppv(0.1, calc_sgy_spec(alg=2, FALSE, FALSE), sens = 0.9)

y2 <- calc_ppv(0.1, calc_sgy_spec(alg=2, TRUE, FALSE), sens = 0.9)

plot(xx, yy, type="l", ylim=c(0.95, 1), col=cols[1], lwd=2.5,

xlab="", ylab="Positive predictive value", main="")

abline(h=c(0.99, 1.0), col="grey30", lty=c(2, 1))

mtext("High prev / 2-test", 1, -1.05, adj=0.95, font=2)

title(xlab="Probability of A1 error after A0", line=1.5)

segments(0.0, y1, 0.202, col=cols[2], lwd=1)

segments(0.0, y2, 0.202, col=cols[3], lwd=1)

mtext("D", 3, 0.3, at=-0.06, font=2, cex=1.5)

##

yy <- calc_ppv(0.01, calc_sgy_spec(alg=3, FALSE, a0=TRUE, corr_a0=xx), sens = 0.9)

y1 <- calc_ppv(0.01, calc_sgy_spec(alg=3, FALSE, FALSE), sens = 0.9)

y2 <- calc_ppv(0.01, calc_sgy_spec(alg=3, TRUE, FALSE), sens = 0.9)

plot(xx, yy, type="l", ylim=c(0.9, 1), col=cols[1], lwd=2.5,

xlab="", ylab="Positive predictive value", main="")

abline(h=c(0.99, 1.0), col="grey30", lty=c(2, 1))

mtext("Low prev / 3-test", 1, -1.05, adj=0.95, font=2)

title(xlab="Probability of A1 error after A0", line=1.5)

segments(0.0, y1, 0.202, col=cols[2], lwd=1)

segments(0.0, y2, 0.202, col=cols[3], lwd=1)

mtext("E", 3, 0.3, at=-0.06, font=2, cex=1.5)

##

yy <- calc_ppv(0.04, calc_sgy_spec(alg=2, FALSE, a0=TRUE,

ass_spec = ass_spec2_mw, corr_a0=xx), sens = 0.9)

y1 <- calc_ppv(0.04, calc_sgy_spec(alg=2, FALSE, FALSE, ass_spec = ass_spec2_mw), sens = 0.9)

y2 <- calc_ppv(0.04, calc_sgy_spec(alg=2, TRUE, FALSE, ass_spec = ass_spec2_mw), sens = 0.9)

plot(xx, yy, type="l", ylim=c(0.96, 1), col=cols[1], lwd=2.5,

xlab="", ylab="Positive predictive value", main="")

abline(h=c(0.99, 1.0), col="grey30", lty=c(2, 1))

mtext("Malawi 2017 / 2-test", 1, -1.05, adj=0.95, font=2)

title(xlab="Probability of A1 error after A0", line=1.5)

segments(0.0, y1, 0.202, col=cols[2], lwd=1)

segments(0.0, y2, 0.202, col=cols[3], lwd=1)

mtext("F", 3, 0.3, at=-0.06, font=2, cex=1.5)

###

###

par(mar=c(0, 0, 0, 0))

plot(0, 0, type="n", bty="n", axes=FALSE)

legend("center", c("A0 + Diagnosis", "Diagnosis", "Diagnosis + Verif."), col=c(cols[1:3]), lty=c(1,1,1), lwd=c(4,2,2)/2, cex=1.0, inset=0.01, horiz=TRUE)
